# Metastasis and MAPK Pathways

**DOI:** 10.3390/ijms23073847

**Published:** 2022-03-31

**Authors:** Mateusz Kciuk, Adrianna Gielecińska, Adrianna Budzinska, Mariusz Mojzych, Renata Kontek

**Affiliations:** 1 Doctoral School of Exact and Natural Sciences, University of Lodz, Banacha Street 12/16, 90-237 Lodz, Poland; 2 Department of Molecular Biotechnology and Genetics, Faculty of Biology and Environmental Protection, University of Lodz, 12/16 Banacha St., 90-237 Lodz, Poland; adrianna.gielecinska@edu.uni.lodz.pl (A.G.); renata.kontek@biol.uni.lodz.pl (R.K.); 3 Laboratory of Mitochondrial Biochemistry, Department of Bioenergetics, Faculty of Biology, Adam Mickiewicz University, 61-614 Poznan, Poland; adrianna.budzinska@amu.edu.pl; 4 Department of Chemistry, Siedlce University of Natural Sciences and Humanities, 3 Maja 54, 08-110 Siedlce, Poland; mmojzych@yahoo.com

**Keywords:** cancer, c-JUN N-terminal kinase (JNK), extracellular signal-regulated kinase (ERK), metastasis, mitogen-activated kinases (MAPKs)

## Abstract

Cancer is a leading cause of death worldwide. In many cases, the treatment of the disease is limited due to the metastasis of cells to distant locations of the body through the blood and lymphatic drainage. Most of the anticancer therapeutic options focus mainly on the inhibition of tumor cell growth or the induction of cell death, and do not consider the molecular basis of metastasis. The aim of this work is to provide a comprehensive review focusing on cancer metastasis and the mitogen-activated protein kinase (MAPK) pathway (ERK/JNK/P38 signaling) as a crucial modulator of this process.

## 1. Introduction

The overgrowth of cells in one specific site usually leads to the formation of primary tumors restricted to one location in the body. However, most patients die because of secondary tumors distant from the original tumor formation sites. The process of tumor metastasis is a complex interplay between the molecular characteristics of cancer cells that acquire certain properties (such as the ability to grow in unfavorable conditions, the capacity to spread across the body, or to produce the enzymes that digest the intracellular matrix) and the tumor microenvironment, which is subject to modifications that facilitate the journey of cells through the blood and lymphatic vessels [1]. All of these new capabilities arise from either genetic or epigenetic changes in the DNA of the transformed cells. Only the minority of tumor cells (subclones) acquire those abilities that enable their spread throughout the organism [2,3].

## 2. Cancer Metastasis Overview

The basement membrane (*basal lamina*, BM) is a 100–300 nm highly dynamic, ultrathin, and dense form of the extracellular matrix (ECM) that separates epithelia or endothelia from the connective tissues located beneath [4]. It consists of large glycoproteins and works as an adhesive scaffold providing strong structural support for other tissues. It also plays important role in cell signaling thanks to the presence of binding sites for cell adhesion molecules and ligands for cell surface receptors, including integrins and growth factor receptors, thus regulating cell functions including proliferation, differentiation, angiogenesis, and migration. BM sets apart tissues via adhesions mediated by hemidesmosomes consisting mainly of laminin that allow cell–matrix adhesion through the interaction with cell-surface integrins [5]. Integrins have a broad extracellular domain that interacts with ECM molecules, and an intracellular region that is connected to the cytoskeleton by intracellular focal adhesions (FAs). More than 150 proteins form FAs, including kinases, scaffold, and adaptor proteins. FAs are dynamic structures that assemble, scatter, and recycle during cell migration. The binding of integrins to FAs and ECM molecules not only allows for cell adhesion to the ECM, but also transfers cytoskeletal forces onto the ECM, allowing for cell migration as well as the transmission of signals from the extracellular environment to the intracellular pathways. The transmission is mediated by integrin-activated signaling molecules, such as focal adhesion kinase (FAK), phosphatidylinositol 3-kinase (PI3K), and extracellular signal-regulated kinase 1 (ERK1). Therefore, integrins are involved in adhesion, migration, and invasion. Integrins also aid in the progression of the metastatic process by causing the basement membrane to be proteolytically degraded by the activation of matrix metalloproteinases (MMPs). Furthermore, integrins are involved in the regulation of tumor cell motility through the transforming protein RhoA (RHOA) signaling pathway [6]. 

BMs encompass 60–200 different proteins and the composition of different BMs is greatly varied. However, four major components with different roles can be distinguished: type IV collagen, laminin, nidogen/entactin, and perlecan. Among others, agrin, bamacan, fibulin, fibronectin, type XV collagen, type XVIII collagen, nephronectin, netrins, SPARC/osteonectin/BM40, BM90, papilin, and usherin can be enumerated [4,7]. The detailed information on the composition of the BM can be found elsewhere: [8,9,10,11]. The composition of tumor BM is different than that of physiological BMs and its function is therefore altered. During the development of neoplasia, multistep spatial and temporal BM remodeling occurs [8]. This process is dynamic and involves de novo deposition and the integration of ECM proteins, their self-assembly, or their degradation [7]. 

Benign tumors do not cross the basement membrane, and therefore are unable to spread further. In contrast, metastatic tumor cells overexpress certain proteases, such as MMPs, which cleave the basement membrane components and allow the movement of tumor cells across the basement membrane and ECM. After the dissolution of the BM, cancer cells may enter the stroma. Moreover, local stromal cells, such as tumor-associated macrophages (TAM), may also produce MMPs and facilitate the movement of tumor cells [12]. Elevated MMP activity was observed in many tumor types, including osteosarcoma, in humans. Research indicates the key roles of MMP2 and MMP9 in the process of metastasis [13]. Moreover, the decreased activity of tissue MMP inhibitors is also often observed in metastatic cancers, allowing the enzymes to freely act on the ECM [14,15]. Cathepsins constitute another class of degrading enzymes with elevated activity observed in many cancers. Cathepsins act on many ECM components, including fibronectin, laminoprotein, and proteoglycans [16]. Moreover, the role of urokinase plasminogen activator (uPA) in metastasis was shown. uPA exhibits serine protease activity and is responsible for the conversion of inactive plasminogen to active plasmin. However, its activity is not restricted to one type of substrate as its proteolytic activity towards ECM and BM was found. The role of uPA in the process of metastasis is not only direct, as it activates other proteases including MMPs [17]. Furthermore, during remodeling, growth factors may be released. This includes vascular endothelial growth factor (VEGF), which stimulates the formation of new blood vessels and contributes to the improved access of cancer cells to oxygen and nutrients [7,18,19]. In the process called the invasion–metastasis cascade, tumor cells eventually violate the barrier, cross the blood or lymphatic microvessels, and spread through general circulation to other sites of the body. The invasion of cancer cells into the vessels is termed intravasation. The thickness of the blood vessel is the crucial factor influencing the ease of intravasation. Thus, small blood and lymph vessels are the major targets of cancer cells [20]. After the cells get into the bloodstream, circulating tumor cells (CTCs) encounter unfavorable conditions such as attacks from the immune system. CTCs often connect and travel as aggregates. Cells adhere either by homotypic adhesion (among cancer cells) or heterotypic adhesion (with other host cells, usually platelets). This prevents the CTCs from elimination as a result of the immune system’s activity (especially in the case of CTCs covered with platelets). Blood platelets are also usually a great source of growth factors that stimulate the growth of cancer cells, further enhancing their chance of survival in the bloodstream [21,22]. The loss of interaction with the ECM may however lead to anoikis, a form of cell death in detached anchorage-dependent cells. In addition, tumor cells become exposed to various environmental stresses, including the lack of oxygen or nutrients, the encounter with reactive oxygen species (ROS), and aforementioned insults from the immune response components [21,23]. However, in favorable conditions, some cells may leave the vessels (extravasate) and form micrometastases, which, over time, may colonize foreign tissues leading to the formation of macroscopic metastases. Cells are disseminated by the vascular and lymphatic drainage [24]. The lungs and the liver work as sophisticated filters of CTCs owing to their dense coverage in small vessels [25]. The colonization of cells may also result from the interactions of ligands (or receptors) on the surface of cancer cells and receptors (or ligands) of endothelial cells (this is also called the seed-and-soil hypothesis). According to the hypothesis, distinct cancer cells may exhibit different sets of ligands or receptors that are compatible with the ligands or receptors on the cells of different tissues [26,27,28]. In addition, some tissues may further attract cancer cells owing to the release of individual chemotactic factors [29]. The other mechanism for the colonization of cells in distinct locations assumes that CTCs may locate in a multitude of tissues wherein the growth of clinically significant metastatic cells is probably only in tissues that secrete factors that facilitate proliferation and colonization by cancer cells [30,31]. Many micrometastatic cells may stay dormant yet viable for an extended time [32]. However, during evolution, cancer cells may adapt to the new environment, colonize foreign tissues, and form a tumor. After achieving a critical size of the tumor mass, the cancer cells need to form a network of new blood vessels to further supply the cells with nutrients, oxygen, and growth factors [33,34]. 

## 3. Molecular Basis of Metastasis

In all cases, tumor formation starts with the initiating mutation that confers the unlimited proliferative potential of cells, followed by the development of genetic instability that leads to autonomic transformed cells. However, the oncogenic transformation is not sufficient for cells to acquire the ability to enter systemic circulation and infiltrate distant tissues. Moreover, the cells need to survive in the new environment and colonize the foreign tissue. The genes that contribute to all these events can be classified into several classes: (a) metastasis initiation genes, (b) metastasis progression genes, and (c) metastasis virulence genes. The first class of genes (initiation genes) allows tissue invasion and their dispersion. This class includes various factors that contribute to angiogenesis, cell motility, or invasion. Progression genes, on the other hand, facilitate tumor development at the site of metastasis. Similar to oncogenes, progression genes contribute to tumorigenesis; however, during the metastatic process they play additional, advantageous functions that enable the tissue-specific spread. The classical examples of these include MMPs. Metastasis virulence genes are responsible for the aggressive potential of tumor cells not in the primary site, but in the secondary site. It is, however, often difficult to classify a gene to a specific class of metastasis genes as their functions usually overlap [35]. 

So far, hundreds of genes have been shown to contribute to the invasive potential of cells. Mutations in genes encoding the cellular tumor antigen p53 (TP53), cyclin-dependent kinase inhibitor 2A (CDKN2A), phosphatase and tensin homolog (PTEN), phosphatidylinositol-4,5-bisphosphate 3-kinase catalytic subunit α (PIK3CA), retinoblastoma (RB1), GTPase KRas (KRAS), estrogen receptor (ESR1), MYC proto-oncogene protein (MYC), and serine/threonine-protein kinase B-raf (BRAF) were linked with the tumor metastasis process. However, metastatic research focuses mainly on gene expression changes during the metastatic process, rather than the exploration of specific gene mutations [36]. 

To acquire the abilities needed to form metastases, epithelial stem cells or differentiated epithelial cells must undergo the epithelial–mesenchymal transition (EMT). The whole EMT process is mediated by various transcription factors involved in developmental programs such as twist-related protein 1 (TWIST), zinc finger proteins SNAI1/2 and SLUG, zinc finger E-box-binding homeobox 1/2 (ZEB1/2), forkhead box protein C2 (FOXC2), or paired mesoderm homeobox protein 1 (PRRX1) [37,38]. The expression levels of noncoding RNAs such as microRNAs and long non-coding RNAs have also been found to change during the EMT process, suggesting that they may play a role in the process [39,40,41,42,43]. The epigenetic regulation, chromatin remodeling, alternative splicing, posttranslational modifications, stabilization, and altered subcellular localization of proteins all contribute to the EMT [44]. 

Cadherins are a transmembrane glycoprotein superfamily that mediates the adhesion between homophilic (same type of cells) cell to cell adhesions. More than 20 members of the cadherin molecule family have been identified, each of which is expressed differently depending on the cell type. A non-covalent interaction occurs between two cadherins of the same kind belonging to nearby cells results in their holding together [6]. The loss of E-cadherin is mainly due to genetic mutations in the *CDH1* gene that promote the synthesis of altered protein, *CDH1* epigenetic silencing through promoter methylation, and SRC family kinase-mediated downregulation of the E-cadherin gene, or the inhibition of *CDH1* expression by transcriptional repressors [45,46]. A functional E-cadherin–catenin complex not only stabilizes cell–cell adhesion, but also activates downstream signaling pathways such as RHO GTPases, PI3K, and MAPK [6]. 

During the EMT, cells change polarity and cytoskeleton organization is altered. The downregulation of an epithelial gene expression signature is one of the main hallmarks of the EMT. This includes the diminished expression of cell junction proteins such as vascular endothelial cluster of differentiation 31 (CD31; PECAM1), claudins, occludins, desmoplakin, and plakophylins [47]. While the loss of E-cadherin and cytokeratins is observed in the EMT, the expression of vimentin and neural cell adhesion molecule (NCAM) becomes upregulated. NCAM interacts with the SRC family tyrosine kinase FYN and contributes to cell invasion and migration [47,48]. Furthermore, cells begin to produce fibronectin, an extracellular matrix protein, normally secreted only by mesenchymal cells [49]. Additionally, the expression of N-cadherin may increase the affinity of cancer cells to stromal cells [50,51]. Through connection with α-catenin and β-catenin, N-cadherin attaches to the cytoskeleton and, via p120 catenin, influences receptor tyrosine kinases (RTKs) activity [47]. Reorganization of the cytoskeleton, gain of front–rear polarity, and the dissolution of the epithelial cell-to-cell junctions are therefore prominent hallmarks of the EMT. During the EMT, reorganization of the cortical actin cytoskeleton occurs enabling dynamic cell elongation and motility [52].

Two main mechanisms of cell movement can be distinguished: amoeboid and mesenchymal. The mesenchymal type of movement is dependent on protease (MMPs) activities that enable the movement through the degradation on ECM, while the amoeboid type is a protease-independent process and relies on mechanical forces exerted by the cells on the ECM. Both types of movements are controlled by the signaling pathways of the RHO family of small GTPases that control actin dynamics and their rearrangement during cell migration in response to environmental stimuli. In the mesenchymal type of movement, cells attain a specific elongated spindle-like shape and resemble fibroblasts in shape. RHOA facilitates actin stress fiber formation, whereas Ras-related protein (RAC1) and cell division control protein 42 homolog (CDC42) promote the formation of lamellipodia (network of the actin filaments) and filopodia (rod-like projections of actin fibers) [52]. The activity of Rho GTPases is regulated by guanine nucleotide exchange factors (GEFs), GTPase-activating proteins (GAPs), and guanine nucleotide dissociation inhibitors (GDIs). RHO-associated kinase (ROCK) interacts with formin diaphanous 1 (DIA1) and facilitates actin polymerization. Furthermore, ROCK inactivates myosin-light-chain phosphatase (MLCP), which normally dephosphorylates myosin II light chain (MLC2). The phosphorylation of MLC2 leads to the augmented activity of myosin II ATPase, facilitating its interaction with actin filaments to confer cell contraction. Phosphorylated MLC2 is negatively regulated by myotonic dystrophy kinase-related Cdc42-binding kinase (MRCK). Furthermore, ROCK1 is negatively regulated by RHO-related GTP-binding protein (RHOE). In contrast, 3-phosphoinositide-dependent protein kinase 1 (PDK1) promotes ROCK1-dependent actomyosin filament formation. PI3K is a key regulator of front–rear polarity and is involved in the recruitment of CDC42 and RAC GEFs to the leading edges of the moving cells. RAC and CDC42 regulate actin polymerization via the regulation of Wiskott–Aldrich syndrome protein (WASP) and its interaction with Arp2/3 complex 34 kDa subunit (ARP2/3) [47,52]. 

In the amoeboid type of migration, rounded cells move via constant cycles of expansion and contraction of the cell body. This is allowed by actin and myosin, which localize cortically and contribute to membrane blebbing. Blebbing results from the inflow of cytoplasm. In contrast, in lamellipodia, actin polymerization is the underlying cause of changes and movement. Again, the RHO/ROCK signaling pathway regulates the process of migration [6,52]. In summary, invadopodia, lamellipodia, filopodia, podosomes, and are actin-rich membrane protrusions formed by metastatic cells. To migrate and invade, protrusions use mechanical forces and proteases. They are either the cell’s sensory organelles (filopodia) for signals such as chemoattractants, or the major organelles for cell motility (lamellipodia), or both allowing motility and ECM degradation (invadopodia). Unlike single-cell migration, collectively migrating cells maintain cell–cell connections by continuously expressing adhesion molecules. Cells may travel as sheets, strands, tubes (coordinated invasion), or clusters (cohort migration). Collective cell migration involves force creation to pull or push cells forward or backward. Substrate-binding integrins generate the energy required for motility. Integrins are expressed on the leading edges of cells to form adhesion complexes with ECM components such as fibronectin. ECM attachment stimulates cytoskeletal adaptor proteins, such as cortactin, vinculin, paxillin, and talin. Migrating cells develop membrane protrusions and integrin-mediated focal adhesions connected to the actin cytoskeleton. Cells also express MMPs at their leading edges to split collagen fibers and arrange them in tube-like structures to travel in the cell mass [6].

MMPs constitute a family of 24 endopeptidases that regulate the ECM composition through proteolytic activity. Based on their structure and function, MMPs can be classified into eight groups encompassing MMPs either secreted or membrane-bound. Due to their proteolytic function, the activity of MMPs must be tightly controlled. This is achieved through strictly controlled transcriptional and translational events. Moreover, MMPs are expressed as proenzymes or zymogens that need to be activated by “cysteine switch” mechanisms and autocatalysis reactions in which the enzyme cleaves its prodomain and gains catalytic activity. Furthermore, as previously mentioned, the activity of enzymes is controlled by four tissue inhibitors of metalloproteinases (TIMPs) that impede their enzymatic function [53]. Due to their proteolytic activity, MMPs have been suggested to play a critical role in all of the metastatic steps from the invasion through ECM degradation [54,55,56], angiogenesis [54,57,58], immune evasion [59,60,61,62], the establishment of premetastatic niche [63,64], extravasation [65], to proliferation and survival in the new environment [66,67,68]. Thus, the inhibition of MMPs may represent a new strategy in targeting metastasis. Despite the obvious roles of MMPs in tumor metastasis, continuous efforts fail to implement MMPs inhibitors into the clinic. This is attributed to the difference between murine and human biology, e.g., differences in lifespan, differences in tumor growth and size without metastatic spread, or the use of genetically homogenous cancer cells in the bolus injection compared with the greater heterogenicity of tumor cells in humans. These factors, together with the lack of the specificity of inhibitors and the potential side-effects, have restricted the use of MMP inhibitors in cancer treatment. Moreover, MMPs act early in the development of metastasis; thus, certain actions, such as MMP expression profiling, should be performed in the pre-and peri-metastatic stages. Both antibodies and small-molecule inhibitors targeting MMPs with varied affinity, specificity, and selectivity have been developed [53].

## 4. Tumor-Associated Macrophages (TAMs) and Cancer-Associated Fibroblasts (CAFs)

It is now well established that cancer cell proliferation and metastasis require interaction with the tumor milieu, which is also referred to as the tumor microenvironment (TME). Tumor interactions with ECM components are critical for the EMT and tumor invasion. TME is engaged in all four steps of the metastatic process: adhesion, detachment, migration, and invasion. TME contains tumor stroma cells, immune system effectors, platelets, fibroblasts, endothelial cells, proteases, cytokines, hormones, and other humoral components [6].

Cancer-associated fibroblasts (CAFs) and tumor-associated macrophages (TAMs) and are the primary components of invading stromal cells. CAFs are a heterogeneous cell population. Every tumor type comprises subpopulations of CAFs that are distinct to the tumor and the surrounding tissue. The activation of CAFs is related to the development of a tumor with invasive characteristics. Cancer progression has been linked to TAMs and CAFs, which have been implicated in the RAS and MAPK signaling cascades. Moreover, CAFs promote primary tumor development via the secretion of cytokines such as interleukin 1 (IL-1) [6,69]. 

In contrast, TAMs may promote the secretion of various factors associated with tumor progression and metastasis, including tumor necrosis factor α (TNF-α), IL-6, transforming growth factor α (TGF-α), platelet-derived growth factor receptor (PDGFR), and vascular endothelial growth factor receptor (VEGFR). These factors can typically affect the EMT, encouraging tumor growth and metastasis. For example, cholangiocarcinoma cells recruit CAFs by secreting platelet-derived growth factor D (PDGF-D), boosting cell migration through PDGFR-β, RHO-GTPase, and c-JUN N-terminal kinase (JNK) pathway activation [70]. TAMs can release a wide range of growth factors, proteolytic enzymes, cytokines, and inflammatory mediators when activated by cancer cells. TAMs can promote cancer metastasis via the stimulation of angiogenesis, proliferation, migration, and invasion [71]. 

Gene expression profiles in metastatic cancers resemble those that allow the physiological function of macrophages or stem cells. Many changes that are thought to account for the EMT may also occur in non-metastatic tumors. Accumulating evidence suggests that other mechanisms, such as the alteration of stem cells, may lead to the development of metastatic cancer cells. Particularly, hematopoietic stem cells or yolk sac-derived macrophages comprise a valid source of metastatic cells (myeloid cell hypothesis). There are many similarities between immune cells and metastatic cells, including the ability to intravasate and extravasate from vasculature, the release of pro-angiogenic factors such as EGF, FGF or PDGF, the capability to survive in hypoxic or necrotic conditions, the expression of cathepsins and the downregulation of E-cadherin. Moreover, when inflammation occurs, macrophages can fuse with epithelial cells and exhibit both epithelial and mesenchymal properties further supporting this hypothesis [33]. 

**Figure 1:**
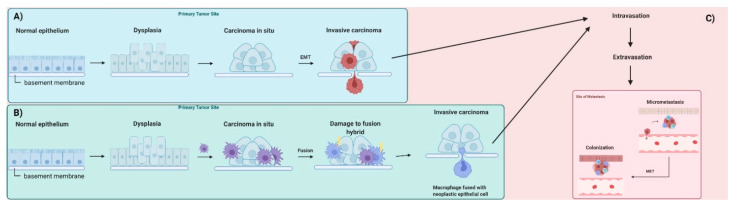
Overview of tumor metastasis. (**A**) Conventional model of metastasis. Normal epithelia are lined with a basement membrane that separates epithelia or endothelia from the connective tissues located beneath. Epithelial cells proliferate and become dysplastic. Accumulation of genetic and epigenetic changes leads to the formation of carcinoma in situ also lined with the basement membrane. During epithelial–mesenchymal transition (EMT), cells acquire abilities that allow them to move through the basement membrane. (**B**) The metastatic process according to the fusion hybrid hypothesis. Metastatic cancer cells are formed as a result of direct transformation or as a result of fusion of neoplastic epithelial cells and myeloid cells such as macrophages. (**C**) Metastatic cancer cells intravasate into small blood (shown here) and lymph vessels, where they travel with circulation to distant sites. At secondary sites, carcinoma cells extravasate, form micrometastases, and in the process of mesenchymal–epithelial transition (MET) colonize the foreign tissue (macrometastasis). Based on [33].

In this hypothesis, macrophage infiltration of carcinoma in situ generates a chronic inflammatory environment that enhances fusion between immune cells and neoplastic epithelial cells. Inflammation leads to the impairment of mitochondrial function and mitochondrial damage, facilitating the use of fermentation as a primary energy source. The emerging hybrids exhibit the properties of mesenchymal cells, similar to those observed for macrophages, such as the ability to intravasate and extravasate. Conventional EMT and the fusion hybrid hypothesis of tumor metastasis are shown in Figure 1 [33]. 

## 5. Cancer Stem Cells in Metastasis

Despite the fact that the presence of cancer stem cells (CSCs) is still widely debated, the CSC hypothesis is of significant clinical significance, because it has the potential to explain tumor resistance to chemotherapy, cancer progression, and recurrence in cancer patients [72,73]. CSCs or tumor-initiating cells (TICs) represent a sub-population of cells that exhibit self-renewal ability and the capacity to differentiate. The undifferentiated state is maintained through the transcriptional regulation of four major transcription factors: SOX2, OCT4, and NANOG [74,75,76,77]. Moreover, compared with normal stem cells, CSCs are highly clonogenic. When a self-renewing stem cell divides, it can choose one of two important cell division fates: symmetric division, which results in the formation of two daughter stem cells; or asymmetric division, which results in the formation of a stem cell and a differentiated daughter cell. Mutations in tumor suppressors that are involved in the restriction of asymmetric stem cell division equip cancer stem cells with mechanisms to hyperproliferate [78,79,80]. Moreover, it was found that cancer cells can undergo the EMT following stimuli from the cells in the tumor environment, which subsequently leads to the formation of cells with features similar to CSCs [81]. Moreover, the conversion of non-stem cancer cells to CSCs, such as in the case of trastuzumab resistance resulting in CSC proliferation, occurs predominantly as a result of IL-6 release by CSCs in the tumor microenvironment. IL-6 is also capable of inducing the expression of other cytokines that are beneficial to CSCs development [82,83,84,85]. Furthermore, through juxtacrine signaling or contact-dependent signaling, TAMs may aid in the formation of a CSC niche [86]. Research indicates that CAFs produce 100 times more interleukin-6 (IL-6) than nonmalignant tissue associated fibroblasts, which can actually enhance the invasive characteristics of the cells [87]. The acquisition of mesenchymal features may help cells to disseminate from primary tumor mass. Likewise, cells may undergo the mesenchymal–epithelial transition (MET) to the epithelial state at the site of metastasis [88,89,90]. 

CSCs emergence is strongly associated with the development of resistance mechanisms to anticancer agents. Chemoresistance of CSCs and their continuous persistence following treatment with anticancer drugs is associated with a high risk of metastasis and the lower survival rates of patients. There are numerous factors that influence the incidence of CSCs resistance. These include interactions of CSCs with microenvironment components, epigenetic alterations occurring in CSCs (disruptions in DNA methylation, nucleosome remodeling, histone modifications, and non-coding RNAs), enhanced drug efflux due to the upregulation of efflux proteins (such as glycoprotein P) and drug inactivating enzymes, enhanced DNA repair and the development of mechanisms that prevent apoptosis and induce quiescence or dormancy [91].

The deregulation of self-renewal pathways implicated in the development of stemness features is considered a hallmark of CSCs. Recent studies have discovered that targeting of these signaling pathways in CSCs is of particular relevance [92]. Despite their importance in embryonic organogenesis and adult homeostasis, the aberrant activation of these pathways promotes a number of features associated with tumorigenesis, including the growth of tumors. These have been extensively reviewed by others, as in the case of Hedgehog [72,93], NOTCH [94,95], and WNT signaling [96,97]. 

Many studies have revealed important connections between CSCs, tumor development, and anticancer therapy resistance, highlighting the need for novel therapeutic options that specifically target this aggressive cancer subpopulation. A variety of surface markers are available today that can be used to recognize CSCs by directly targeting the antigens present in these cells. When compared to non-neoplastic stem cells and somatic cells, the expression profiles and sub-localizations of these markers in cancer stem cells are significantly different. Apart from molecular markers, various of analytical procedures and techniques are utilized to identify cancer stem cells. These methods and techniques range from functional tests to cell sorting and filtration strategies or xenotransplantation methods [98]. Several approaches based on CSC targeting may be effective in improving clinical responses to systemic therapy. These rely on CSC ablation with monoclonal antibodies, the inhibition of CSC function, the reversal of CSC-associated resistance mechanisms, and the induction of CSC differentiation with epigenetic differentiation therapy [99]. Small molecule inhibitors, vaccines, antibodies, and chimeric antigen receptor T cell cells (CAR-Ts), are currently being tested in clinical trials to assess their effectiveness and safety [81,100].

## 6. Major Metabolic Adaptations of Metastatic Cells

As previously mentioned, wandering cells may experience unfavorable conditions such as hypoxia. Thus, cells must undergo metabolic adaptations to survive in low-oxygen environments. One of the crucial factors that determine survival in hypoxic conditions is hypoxia-inducible transcription factor α (HIF1α), which works as a transcription factor for a number of genes involved in anaerobic metabolisms, angiogenesis, and metastasis [35,46]. While the HIFα subunit is targeted for degradation by the von Hippel–Lindau tumor suppressor (VHL) under normoxic conditions, the occurrence of hypoxia results in the translocation of the HIFα subunit to the nucleus and its association with constitutively expressed HIFβ subunit, which results in the activation of expression of various genes containing a HIF-responsive element (HRE). These include carbonic anhydrases, glucose transporters, and VEGF [35,46,101,102]. Additional studies have shown that VEGF promotes endothelial cell survival by activating the phosphotyrosine 3-kinase/RAC-alpha serine/threonine-protein kinase (PI3Ks/AKT) signal transduction pathway and increasing the expression of the anti-apoptotic protein BCL-2 [103].

**Figure 2:**
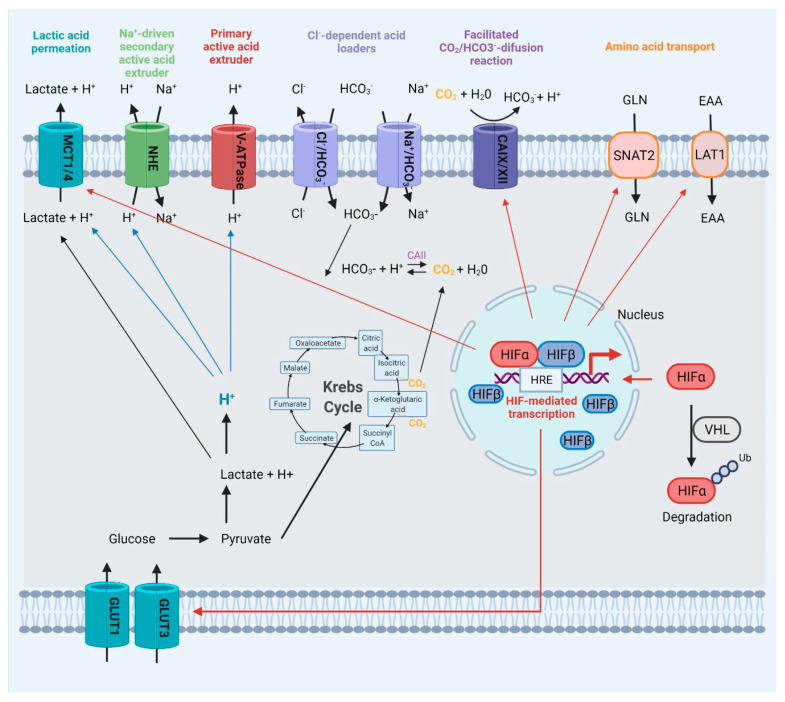
Major metabolic adaptations of cancer cells during metastasis with special focus on ways by which cancer cells avoid repercussions resulting from hypoxia and lack of nutrients. Hypoxia-inducible transcription factor α (HIF1α) is a transcription factor for many genes involved in anaerobic metabolisms, angiogenesis, and metastasis. HIFα subunit is targeted for degradation by the von Hippel–Lindau tumor suppressor (VHL) under normoxic conditions; however, the occurrence of hypoxia allows the translocation of the HIFα subunit to the nucleus and formation of transcription factor complexes with constitutively expressed HIFβ subunit. The transcription factor regulates the expression of various genes containing HIF-responsive elements (HRE) such as carbonic anhydrases, glucose transporters, and vascular endothelial growth factor (VEGF). MCT1–MCT4 are proton-coupled monocarboxylate (lactate, pyruvate, and ketone) transporters. Sodium hydrogen ion exchangers (NHE) regulate the intracellular pH regulation via electroneutral, 1:1, exchange of Na^+^ and H^+^ along their gradients. Vacuolar-type H^+^ adenosine triphosphatases (V-ATPases) transport H^+^ across membranes in an active process. Carbonic anhydrases (CA) are zinc metalloproteins that catalyze the reversible hydration of CO_2_ to form HCO_3_^−^ and H^+^. CO_2_ is also the main byproduct of the Krebs cycle and may be generated from HCO_3_^−^ in reactions catalyzed by CAII. HIF transcription factor governs the expression of MCT1/4, glucose transporters (GLUT1/3), amino acid transporters—sodium-coupled neutral amino acid transporter 2 (SNAT2), large neutral amino acid transporter 1 (LAT1), and CAIX/CAXII. Cl^−^/HCO_3_^−^—chloride bicarbonate anion exchangers; Na^+^/HCO_3_^−^—sodium-dependent bicarbonate cotransporters.

Restricted access to oxygen supply implies heavy energetic reliance of cancer cells on lactate produced in anaerobic respiration [104,105]. Cancer cells have been found to overexpress the monocarboxylate transporters 1 and 4 (MCT1 and MCT4), as well as the glucose transporters 1 to 3 (GLUT1–GLUT3), all of which contribute to cell survival under stress conditions. MCTs are responsible for the transport of monocarboxylic acids (such as lactate, pyruvate, and ketone bodies) into and out of cells through the plasma and mitochondrial membranes. These proteins have a fundamental function in regulating the efflux of lactate and protons produced as byproducts of glycolysis from the intracellular to extracellular space. As a result, they contribute to extracellular acidosis. GLUT1–3, on the other hand, control the uptake of glucose by the cells, which is then transformed to pyruvate, resulting in the production of two ATP molecules. The cytoplasmic pH is regulated by five primary protein families. H+ transporters, such as MCT transporters, sodium hydrogen ion exchangers (NHE), as well as vacuolar-type H+-adenosine triphosphatases (V-ATPases) that transport H+ across membranes, and chloride–bicarbonate exchangers. The sodium-coupled bicarbonate transporters (SLC4 and SLC6) control the intake of bicarbonate used to titrate intracellular H+. Furthermore, carbonic anhydrases are responsible for pH regulation by catalyzing the reversible hydration of CO_2_ [106,107,108,109,110] (Figure 2). 

Furthermore, glutamine absorption and glutaminolysis are critical for the survival of some tumor cells. Carbon used for the tricarboxylic acid cycle, glutathione production, and nucleotide and lipid synthesis is provided from glutamine via reductive carboxylation. Moreover, glutamine is essential for the supply of reduced nitrogen for biosynthetic pathways. Even though mammalian cells are capable of glutamine production due to the activity of glutamine synthetase (GLUL), some cancer cells require exogenous glutamine, which is catabolized in the mitochondria by another enzyme—glutaminase (GLS) [111]. HIF transcription factor regulates the expression of amino acid transporters—sodium-coupled neutral amino acid transporter 2 (SNAT2) and large neutral amino acid transporter 1 (LAT1) engaged in the transport of glutamine for enhanced growth [112] (Figure 2).

Additionally, CSCs were shown to rewire the energy metabolism to meet the demands of self-renewal and stemness maintenance. Growing data support the fact that changes in lipid metabolism, such as an increase in fatty acid (FA) consumption, de novo lipogenesis, and the stimulation of mitochondrial FA oxidation, are involved in the control of CSC function. The role of lipid metabolism in CSCs was recently reviewed by other authors [113,114].

Cell detachment triggers anoikis, characterized by the simultaneous downregulation of anti-apoptotic protein BCL-xL and the upregulation of FAS ligand that works as a death receptor pathway activator. Therefore, CTCs incorporate mechanisms to avoid anoikis and apoptotic cell death during metastasis. This includes the downregulation of caspase 8 (CASP8) that promotes cell dissemination and survival, and alterations in the signaling pathways that regulate anoikis including small GTPases, various protein kinases, and EMT factors [35,115].

## 7. The Signaling Pathways in Metastasis

RTKs play a pivotal role in the regulation of various cellular activities including differentiation proliferation, and migration. The ErbB family of receptors includes four receptors, ErbB-1 (EGFR), ErbB-2 (HER2 or NEU), ErbB-3, and ErbB-4. Similar to other receptor tyrosine kinases, epidermal growth factor receptor (EGFR) is composed of extracellular, transmembrane, and intracellular regions. The extracellular region contains domains responsible for ligand binding. Many EGFR ligands have been identified so far. These include amphiregulin, betacellulin, epidermal growth factor (EGF), epiregulin, and transforming growth factor α (TGF-α). Some of them bind directly with the receptor, while others need to be processed in proteolytic cleavage reactions that allow their effective binding [116,117]. On the other hand, the transmembrane domain anchors the receptor in the phospholipid bilayer of the cell, while the intracellular domain possesses catalytic activity. The binding of the ligand triggers homo- or hetero-dimerization with other ErbB family members and leads to subsequent transphosphorylation of intracellular domains, which generates the binding sites for many adaptor proteins involved in signaling events. EGFR receptors form complicated signaling networks with other signaling pathway components including proteins involved in the mitogen-activated kinase MAPK (RAS/RAF/MAPK), phosphatidylinositol 3-kinase (PI3K/AKT), protein kinase C (PKC), and the JAK/STAT pathways. Moreover, EGFR has been shown to translocate to the nucleus, where it controls the expression of various genes including *CCND1*, coding for cyclin D1 involved in cell cycle progression. For example, EGFR is overexpressed in 80–90% of aggressive head and neck squamous cell carcinomas and is associated with poor survival [118,119]. 

Several studies have demonstrated that the expression levels of EGF and EGFR are associated with tumor progression, metastasis formation, and resistance to treatment [120]. EGF may play an important role in the acquisition of metastatic properties in gallbladder cancer (GBC). Sasaki et al. reported that EGF may promote the EMT and the development of stemness in GBC cells with a scattering phenotype by increasing the activity of β-catenin in these cells [121]. Experiments on human colon cancer cells in vitro have revealed that metastatic cells may express up to five times more EGFR than nonmetastatic cells. This influences the aggressiveness of the tumor cells [122]. Moreover, research indicates the intrinsic relationship between transforming growth factor α (TGF*α*) and EGFR [123]. The findings provide evidence for the notion that some cells may rely on TGF-induced EGFR activation and promote IL-8 production and, thus, together with VEGF, stimulate the formation of new blood vessels to enhance the survival of cancer cells and promote their metastatic potential [124]. Since Virchow’s hypothesis, the complex link between cancer and chronic inflammation has been carefully studied. Through the years it was established that inflammation regulates cancer progression [125]. IL-8 and VEGF are both expressed at high levels in a variety of tumors, and they have been shown to enhance tumor angiogenesis, growth, and metastasis. To promote the creation of new blood vessels, ERK1/2 can be employed as an alternate mechanism to stimulate the expression of IL-8 and VEGF. Moreover, it has been shown that the activation of the ERK/MAPK signaling pathway is involved in the promotion of VEGF production in colorectal cancer [126]. VEGF-C contributes to enhanced skin cancer migration, invasion, and stemness via upregulation of SLUG transcription factor in KRAS/MAPK-dependent signaling events [127]. Several members of the MMP family, especially MMP2 and MMP9, were shown to be highly expressed in tumors that were positive for TGF*α* [124]. Moreover, the expression of TGF*α* may be the major determinant of response to treatment with EGFR inhibitors [128,129]. Many studies were performed to determine the efficiency of dual inhibition of EGFR and VEGFR. Experiments in murine models have shown that the treatment is effective and reduce both the growth and metastasis in nude mice model of orthotopic human colon cancer [130,131]. Moreover, the use of combination therapy with EGFR, VEGFR [132], and PDGFR inhibitors together with gemcitabine constitutes promising therapy for pancreatic cancer with strong (80–95%) inhibition of tumor growth and protracted survival in the orthotopic nude mouse model [132,133]. Dual inhibition of EGFR and VEGFR was also tested in colon cancer [134], cutaneous squamous cell carcinoma [135], follicular thyroid cancer [136], oral cancer [137], orthotopic ovarian carcinoma [138], and prostate cancer [139,140]. EGFR is currently a key target in anticancer drug discovery. As a result, monoclonal antibodies such cetuximab, panitumumab, nimotuzumab, necitumumab, and others have been introduced into the clinic. There are two basic techniques to combine chemotherapeutic agent toxicity and the precise targeting of EGFR overexpressing tumor tissues: antibody–drug conjugates (ADCs) and antibody–nanoparticle conjugates (ANCs). ADCs are antibodies covalently attached to a cytotoxic agent via a spacer. ADCs undergo chemical and enzymatic processing, resulting in the release of the drug upon binding to EGFR, and endocytosis. ANCs are nanotechnology-based formulations and include lipid, polymeric, and inorganic nanoparticles that preserve drugs against inactivation, allowing regulated release and passive accumulation in tumor tissues through increased permeability and retention. EGFR receptor-mediated endocytosis and the formation of lysosomes results in the release of drug into the cytosol. This topic was reviewed previously in [141]. EGFR is the most well-known growth factor receptor that interacts with G protein-coupled receptors (GPCRs) and is involved in the regulation of tumor development, invasion, and progression in a variety of malignancies [142]. 

The class of surface receptor proteins known as GPCRs regulates a wide range of cellular functions and has been identified as a potential treatment target for several diseases including cancer. GPCRs are activated by hormones, neurotransmitters, growth factors, or light. Additionally, GPCR ligands such as prostaglandins, bradykinin, and gastrin-releasing peptide can also transactivate EGFR to promote cancer cell proliferation, survival, and invasion. PI3K/AKT, PDK1, and MMPs are some of the essential signaling intermediates involved in GPCR–EGFR crosstalk [142,143,144]. 

Instead of a single G protein component such as RAS, G protein-coupled GPCRs have three G protein subunits that attach to the plasma membrane via Gα and Gγ subunits. The Gα subunit also binds to GTP (active protein) or GDP (inactive protein) upon activation of GPCR. Heterotrimeric G proteins dissociate into Gα monomers and Gβ–Gγ dimers, relaying the message to downstream signaling partners. Through the modulation of several biological pathways, they have the potential to either suppress or accelerate tumor development, survival, dissemination, and metastasis. GPCRs may also upregulate mesenchymal transcription factors such as the SNAI, ZEB, and TWIST superfamilies, which regulate cell polarity, cytoskeleton remodeling, migration, and invasion, through modulation of downstream signaling pathways such as the nuclear factor kappa B subunit (NF-κB), MAPK/ERK, PI3K/AKT, and TGF-β pathways [142]. 

The transforming growth factor-β (TGFβ) family constitutes a group of homo- and heterodimeric secreted cytokines that control the multitude of cellular functions including proliferation and differentiation of cells. All cell types express TGF-β receptors responsible for signal transduction upon ligand binding. Type I and II receptors transduce signals through their intrinsic ATP-dependent protein kinase activity. Upon ligand binding, constitutively active type II receptor (TβRII) recruits TGF-β type I receptor (TβRI). TβRII activates the TβR1 via phosphorylation of its regulatory segment called the GS region. The binding of the ligand leads to the recruitment and phosphorylation of mothers against decapentaplegic (SMAD) family proteins: receptor-activated SMADs (R-SMADs) and receptor-regulated SMADs—SMAD2 and SMAD3. R-SMADs associate with SMAD4 and SMAD2/3-SMAD4 complexes, translocate to the nucleus, and act as transcription factors. The whole process is tightly controlled by SMAD6 and SMAD7, which are also called inhibitory SMADs (I-SMADs). However, as with the other signaling pathways discussed in this review, the complexity of the regulation of signal transduction is beyond the scope of this work. Thus, we refer the reader to other reviews on this topic [145]. 

**Figure 3:**
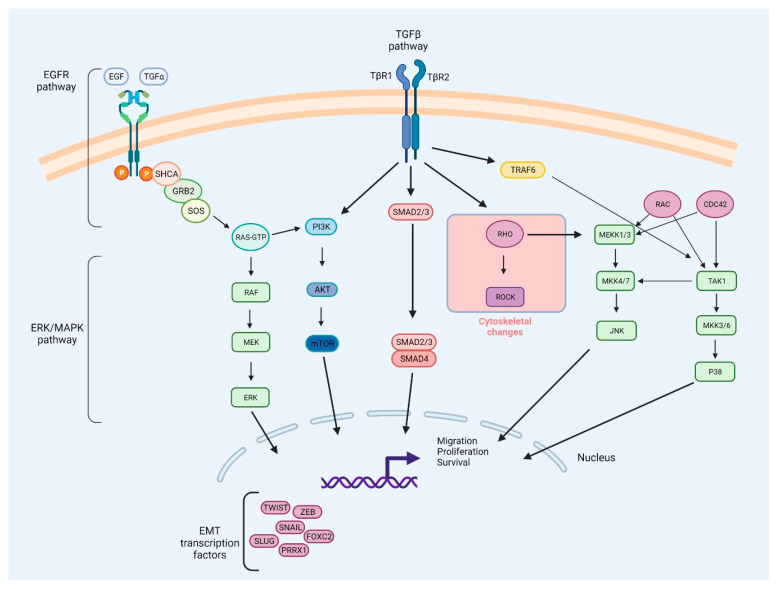
Integration network of epidermal growth factor receptor (EGFR), mitogen-activated protein kinase (ERK/MAPK), and transforming growth factor-β (TGFβ) signaling pathways involved in metastasis processes. Several signaling pathways are involved in the progress of the epithelial–mesenchymal transition (EMT), and these pathways can work together to elicit complete EMT responses. Apart from its ability to promote EMT through the expression of mothers against decapentaplegic (SMAD) proteins, transforming growth factor β (TGF-β) can also activate the phosphotyrosine 3-kinase/RAC-alpha serine/threonine-protein kinase (PI3K–AKT), ERK–MAPK, P38–MAPK, and c-JUN N-terminal kinase (JNK) signaling pathways. After TβRI phosphorylates the adaptor protein SRC homology 2 domain-containing-transforming A (SHCA), it provides a docking site for the growth factor receptor-bound protein 2 (GRB2) and the son of sevenless (SOS), an event that signals the initiation of the MAPK cascade involving RAS, RAF, MEK, and ERK. Interaction of TNF receptor-associated factor 6 (TRAF6) with the TGF receptor complex stimulates TGF-activated kinase 1 (TAK1), which contributes to activation of P38 and JNK. The EMT is facilitated by the activation of ERK1 and ERK2 MAPK, which increase the expression of EMT transcription factors and proteins involved in cell motility or invasion, such as RHO GTPases that activate the RHO-associated kinase (ROCK) protein to confer cell contraction. Hypoxia-inducible factor 1 (HIF1) (not shown here) can promote EMT by activating the expression of the TWIST protein, which acts as a transcription factor. TGFβ signaling involves activation of SMAD proteins that stimulates expression SNAI/L and down-regulates the expression of VE-cadherin, CD31, and claudin 5 to facilitate the metastasis process. Other EMT-inducing transcription factors include forkhead box protein C2 (FOXC2), paired mesoderm homeobox protein 1 (PRRX1), SLUG, and zinc finger E-box-binding homeobox (ZEB). Furthermore, the PI3K–AKT signaling pathway may be activated both by EGFR and TGFβ signaling.

TGFβ signaling plays a dualistic role in cancer. This is due to a versatility of function of TGFβ signaling, such as the influence on ECM remodeling, control of tumor cell interaction with the microenvironment, transcriptional regulation, or angiogenesis. On the one hand, due to the SMAD-dependent downregulation of c-MYC, cyclin-dependent kinases (CDKs), and M-phase inducer phosphatase 1 CDC25A (CDC25A), TGFβ may restrict the proliferation of cells [146]. On the other hand, it may act as a tumor growth and metastasis promoter. This turnover is complex, but may be attributed to the phosphoserine/phosphothreonine-binding protein 14-3-3ζ that induces the tumorigenic activity of TGF-β. It emerged that 14-3-3ζ may stabilize glioma-associated oncogene homolog 2 (GLI2), a partner of SMAD complexes, and induce the expression of the parathyroid hormone-related protein (PTHrP) which, together with TNF-α and cytokines, stimulates osteoblasts to release tumor necrosis factor ligand superfamily member 11 (RANKL). RANKL works as a ligand for the tumor necrosis factor superfamily member 11 (RANK), which enhances osteoclast differentiation, further supporting bone demineralization and release of factors that enhance cell proliferation [46,147]. Moreover, the activation of TGFβ stimulates the expression of SNAI and downregulates the expression of VE-cadherin, CD31, and claudin 5, which facilitates the metastasis process [44]. TGFβ may also stimulate AKT activity through PI3K in epithelial cells undergoing the EMT, which leads to the activation of the mammalian serine/threonine-protein kinase TOR complexes 1/2 (mTORC1 and mTORC2) [148,149,150]. TGFβ increases mTORC2 kinase activity in cells undergoing the EMT, regulating the advancement of epithelial cells through the EMT process. mTORC2 is necessary for cell migration and invasion because it regulates the cytoskeletal alterations and gene expression that occur in the course of EMT [150]. MAPK pathways, such as ERK, P38, and JNK, are also activated by TGFβ signaling [151]. Furthermore, TGFβ-induced transcription is enhanced by ERK/MAPK signaling, which results in a greater reduction in E-cadherin expression and increased expression of N-cadherin and MMPs [47,152]. The integrated network of the EGFR, ERK/MAPK, and TGFβ signaling pathways involved in the metastasis process is presented in Figure 3.

## 8. The MAPK Pathway in Metastasis

**Figure 4:**
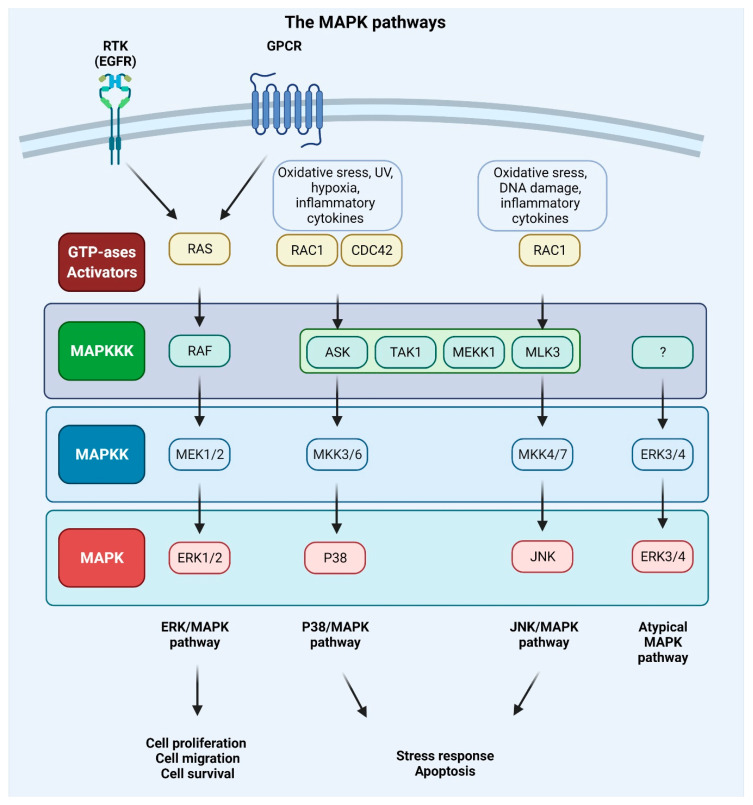
Mitogen-activated protein kinase (MAPK) signaling pathways. Extracellular signals such as growth factors and cytokines, as well as intracellular signals such as oxidative and DNA damage, activate MAPK pathways. GTPases (activators) such as RAS, RAS-related protein (RAC), and cell division control protein 42 homolog (CDC42) constitute the first layer of MAPK signaling cascade, which conveys the signal to downstream protein kinases. The MAPK signaling cascades consist of three kinases: mitogen-activated protein kinase kinase kinase (MAPKKK), a mitogen-activated protein kinase kinase (MAPKK), and mitogen-activated protein kinase (MAPK) and results in proliferation, migration, differentiation, survival, or apoptosis. Mammalian MAPK pathways include ERK MAPK, P38 MAPK, and JNK MAPK signaling events. ERK/MAPK pathway is activated by RAS, which is attracted to the plasma membrane through receptor tyrosine kinases (RTKs) and G protein-coupled receptor (GPCRs) activation. In this cascade, MAPK/ERK kinase 1/2 (MEK1/2) activates extracellular signal-regulated kinase 1/2 (ERK1/2). P38/MAPK and JNK/MAPK pathways are triggered by various insults that activate signaling through MKK3/6 or MKK4/7 (MAPKKs), respectively, that are activated upon MAPKKK s such apoptosis signal-regulating kinase 1 (ASK1), transforming growth factor-β-activated kinase 1 (TAK1), MEKK1 (MAPKKK), and MLK3 (MAPKKK). ERK3/4 are considered atypical MAPK kinases [156].

MAPKs are serine/threonine-protein kinases that can be activated by a variety of extracellular stimuli including growth factors, cytokines, insulin, environmental factors, and oxidative and genotoxic stress. Through the use of genetically engineered mouse models and chemically induced tumorigenesis models it has been observed that components of the MAPK pathway not only regulate the behavior of tumor cells, but also the behavior of surrounding normal stromal cells in the TME during cancer pathogenesis. The unique activities of MAPK pathway components in tumor initiation and development vary based on the stimuli and stromal cells involved in tumor growth, as well as the molecular isoforms of the pathway components, as reviewed in [153,154]. The conventional MAPKs in mammals include c-Jun NH2-terminal kinase (JNK), P38 MAPK, and extracellular signal-regulated kinase (ERK), which, in turn, exist in several isoforms. JNK1 and JNK2 are found in nearly all tissues in contrast to JNK3, which is found primarily in neuronal cells. P38 MAPK exists in several isoforms encompassing P38α (also known as MAPK14 or SAPK2a), P38β (MAPK11, SAPK2b), P38γ (MAPK12, SAPK3, ERK6), and P38δ (MAPK13, SAPK4). ERK1 and ERK2 are the subtypes of the eight isoforms of ERK that are activated by MAPK/ERK kinase 1 (MEK1/2) [155,156]. In contrast, ERK3/4 and ERK7/8 are considered atypical MAPKs [157]. The MAPK pathway components are shown in Figure 4. 

Every MAPK signaling cascade involves at least three core kinases: MAPKKK (mitogen-activated protein kinase kinase kinase), MAPKK (mitogen-activated protein kinase kinase), and MAPK (mitogen-activated protein kinase) [158]. MAPK pathways are present in nearly all eukaryotes and play an important role in numerous cellular activities, including gene expression, metabolism, proliferation, apoptosis, invasion, and metastasis [156]. The RAS–RAF–MEK–ERK pathway is disrupted in approximately 40% of all human malignancies, with mutations in BRAF (10%) and its upstream activator RAS (30%) being the most frequently observed. Inhibitors of MEK were among the first anti-MAPK pathway therapeutics to be created, but despite their high potency and selectivity, they failed in clinical trials. This was due to the negative feedback mechanisms of the pathway components and systemic toxicity of the drugs. Treating metastatic malignant melanoma and other cancers with the use of a combination of RAF and MEK inhibitors has become standard practice. The majority of the pioneering work has been performed in metastatic malignant melanoma, which is characterized by a high prevalence of BRAF (50–60%) and NRAS (15–20%) mutations [159,160,161,162,163]. 

The MAPK pathway is initiated by the binding of growth factor to the RTK or GPCR and the subsequent phosphorylation of RAS protein and following activation of BRAF or RAF (also known as MAPKKK) kinase. The activation signal is conveyed to the MAPKKs (phosphorylation of two serine residues of the MEK1 or MEK2 protein). The downstream phosphorylation of tyrosine and threonine residues of ERK kinase results in the phosphorylation of a multitude of protein substrates involved in differentiation, apoptosis, and migration [41,156]. Upon stimulation of MAPK signaling, ERK1/2 shuttles from cytoplasm to the nucleus, where it regulates gene expression by phosphorylating numerous transcription factors. In the cytoplasm, cytoskeletal components such as microtubule-associated protein (MAP1, MAP2, MAP4) are the targets of the ERK1/2 kinase. These phosphorylation events control the cell morphology and cytoskeletal redistribution. Moreover, ERK1/2 may phosphorylate other cytoplasmic components, including son of sevenless (SOS), RAF1, and MEK, providing a negative feedback regulation of the pathway. In the nucleus, proto-oncogenes including c-FOS, c-JUN, ETS domain-containing protein (ELK1), c-MYC, and cyclic AMP-dependent transcription factor (ATF2) are all phosphorylated in an ERK1/2-dependent manner [126]. The activation of the ERK/MAPK signaling pathway can promote tumor invasion and metastasis by upregulating MMP expression, whereas the inhibition of this signaling can impede the aforementioned processes [164,165]. It was also discovered that mesothelin regulates the expression of MMP7 through the MAPK/ERK signal transduction pathway, as well as the ERK1/2, AKT, and JNK-mediated pathways, contributing to the invasiveness of ovarian cancer cells [166]. Moreover, RAS-associated protein RAP1A was identified as a significant promoter of ovarian cancer cell metastasis via activation of ERK and P38 signaling and the induction of EMT through enhanced expression of SLUG, ZEB1, vimentin, fibronectin, and MMP9 [167]. 

The modification of cellular adhesiveness has a direct impact on the mobility of cells. The activation of the ERK/MAPK pathway has been demonstrated to regulate the disassembly of focal adhesions [158]. Moreover, fibroblast de-adhesion triggered by EGFR necessitates the activation of M-calpain, which is downstream of the ERK/MAPK kinase signaling pathway [168,169,170,171]. Previous studies indicate that activation of the MAPK pathway may not be sufficient for the induction of cell mobility and may require phospholipase C activity (PLC) [172,173]. Moreover, the RAS/RAF/MEK/ERK kinase cascade can have a profound impact on HIF-1*α* protein translation. Activated ERK phosphorylates eukaryotic translation initiation factor 4E-binding protein 1 (4E-BP1), ribosomal protein S6 kinase (S6K), and MAP kinase interacting kinase (MNK) (which can, in turn, directly phosphorylate eukaryotic translation initiation factor 4E (eIF-4E)) and enhances mRNA translation of HIF-1*α* protein involved in the response of cells to hypoxia [174]. Moreover, ERK may alter MMP activity, which affects gastric cancer (GC) cell migration or invasion, and many proteins upstream of the ERK/MAPK pathway, such as IL-22, RasGAP-activating-like protein 1 (RASAL1), protein tyrosine phosphatase type IVA 3 (PRL3), nuclear apoptosis-inducing factor 1 (NAIF1), coiled-coil domain-containing protein 134 (CCDC134), and zinc finger protein (ZIC1) that potentially affect invasion and migration in GC cell lines [158]. The RAS/RAF/ERK cell signaling pathway and the P38 MAPK pathway are both responsible for the activation of MNKs that are engaged in oncogenic transformation and can promote metastasis. Alternative splicing results in the production of four MNKs isoforms in human cells (MNK1a/b and MNK2a/b), which are derived from two genes. Through the regulation of eukaryotic translation initiation factor 4E (eIF4E), these kinases play a critical role in the control of the expression of specific proteins involved in the cell cycle, cell survival, and cell motility. However, they also regulate the expression of genes through the modulation of other substrates such as heterogeneous nuclear ribonucleoprotein A1 (HNRNPA1), polypyridine tract-binding protein-associated splicing factor (SFPQ), and sprouty 2 (SPRY2). This topic was recently reviewed in [175].

ROS play a critical role in the regulation of various biological processes. ROS are an integral part of the tumor microenvironment and may promote cancer angiogenesis, metastasis, and survival. Several studies have demonstrated that ROS accumulation is a significant contributor to the EMT process and this topic has been previously reviewed [176,177,178]. For example, ROS causes epigenetic alterations in the promoter region of E-cadherin and several other tumor suppressor genes, resulting in tumor development and metastasis. It may cause gene promotor hypermethylation via SNAI-mediated induction of histone deacetylase 1 (HDAC1) and DNA methyltransferase 1 (DNMT1). Moreover, it was found that TGF-β1 controls the expression of uPA and MMP9, which aids in cell motility and invasion through ROS-mediated events. ROS present in moderate concentrations, stimulate the activation of the cancer cell survival signaling cascade, which includes the MAPK/ERK, P38, JNK, and PI3K/AKT signaling. As a result of the pathway activation ROS contribute to the activation of NF-κB, MMPs, and VEGF. However, cells have to maintain a balance between ROS generation and elimination, as excess ROS production may lead to DNA damage and apoptosis [125]. Moreover, EGFR/RAS/MAPK signaling pathway is involved in NFκB activation, cyclooxygenase-2 (COX2) upregulation, and GC cell proliferation. COX2 upregulation promotes cancer growth and decreases apoptosis. Many studies on GC have linked the MAPK pathway activation to apoptosis and autophagy. The role of the MAPK pathway in GC was extensively reviewed in [42].

The P38 pathway includes the MAPKKKs such as apoptosis signal-regulating kinase 1 (ASK1), transforming growth factor-β-activated kinase 1 (TAK1), mitogen-activated protein kinase kinase kinase 1 (MEKK1), and mixed-lineage kinase 3 (MLK3), and MAPKKs, such as MKK3/6, which in turn activate P38 [70]. In various malignancies, P38 promotes EMT and metastasis via the upregulation of pro-metastatic genes [179,180]. P38 MAPKs perform a wide range of functions through binding to and activating a diverse array of substrates. More than 100 proteins have been demonstrated to be susceptible to direct phosphorylation by P38 MAPKs in vitro and in vivo, with approximately half of these being transcription factors, including ATF-1, -2, and -6, TP53, and CCAAT/enhancer-binding protein α (C/EBPα). Other substrates include protein kinases (e.g., MAP kinase-activated protein kinase 2/3 (MK2/3), ribosomal protein S6 kinase α 5 (MSK1) and phosphatases (e.g., serine/threonine-protein phosphatase 2A catalytic subunit α isoform (PPP2CA)), cell-cycle proteins (e.g., cyclin D1), apoptosis proteins (e.g., BCL-2 family proteins), growth factor receptors (e.g., fibroblast growth factor receptor 1 (FGFR1)), and cytoskeletal proteins (e.g., tau, keratin 8) [181]. For more information on substrates of P38 see [182,183]; moreover the table, a companion to the SnapShot “p38 MAPK Signaling” in the January 31 issue of *Cell*, describes 66 P38α substrates grouped into eight different categories based on biochemical function [184]. In this review, we will focus on P38 targets with an established role in cancer metastasis.

It has been demonstrated that P38 may be involved in the phosphorylation of the Ser68 residue on TWIST1, which leads to increased protein stability and promotes its capacity to induce EMT and invasiveness in breast cancer [185]. It has been found that elevated TWIST1 levels are also dependent on activation of the ERK signaling [186]. TWIST1 may in turn act as a transcriptional factor for MMPs [187]. Additionally, P38-mediated signaling was shown to regulate the expression of MMP1, MMP2, MMP9, and MMP13 in multiple cancer cell lines [188,189,190,191,192]. In contrast, high expression levels of SNAI together with high expression levels of the phosphorylated P38 MAPK (Thr180/Tyr182) were found in primary tumors. High expression of SNAI in metastatic cells is correlated with an increased risk of death in ovarian cancer patients [193]. A strong link between inflammation and EMT has been established [194]. SNAI was found to trigger IL-6 production, which may in turn act as an EMT trigger. IL-6 also contributes to signal transducer and activator of transcription 3 (STAT3) activation, which affects both tumorigenesis and metastasis [195,196,197]. IL-1β is another cytokine linked to the advancement of cancer including gastric adenocarcinoma, but its molecular causes remain unknown. Both P38 and JNK regulate the IL-1β signaling pathway and the activation of P38 by IL-1β enhances GC cell motility, invasion, and metastatic potential in vitro and in vivo. It was shown that IL-1β induces the IL-1β/P38/AP-1(c-FOS)/MMP2/MMP9 pathway [198]. 

The Forkhead box (FOX) family of transcription factors, which are distinguished by a conserved DNA-binding domain known as the ‘forkhead’ or ‘winged-helix’, regulate a wide range of biological functions, including cell proliferation, differentiation, apoptosis, and metabolism. FOXC1 and FOXC2, play a critical role in the regulation of embryonic, ocular, and cardiac development. A wide variety of cancers are including breast carcinomas, hepatocellular carcinomas, lymphomas exhibit elevated expression of FOXC1 and FOXC2. FOXC transcription factors aid in the progression of cancer through regulation of cell proliferation, metastasis, EMT, and angiogenesis [199]. FOXC1 promotes tumor metastasis in numerous human malignant cancers. However, the upstream and downstream molecular mechanisms of FOXC1 in metastasis remain unknown. FOXC1 upregulation was related to poor prognosis in colorectal cancer (CRC). In vitro and in vivo, FOXC1 knockdown reduced CRC cell migration and invasion while FOXC1 overexpression increased the metastatic potential of the tested cells. Moreover, it was found that in metastatic CRC cells, FOXC1 regulates MMP10 and the expression of transcription factors SOX4 and SOX13. FOXC1’s Ser241 and Ser272 were found to be important sites for the interaction with P38, phosphorylation of which contribute to its stability [200]. Moreover, the P38-mediated phosphorylation of Ser367 of FOXC2 serves as a regulatory mechanism of ZEB1 in metastatic breast cancer cells. The inhibition of P38–FOXC2 signaling selectively reduces cell metastasis without an effect on primary tumor growth. The genetic or pharmacological suppression of P38 reverses the EMT in a FOXC2-dependent process [201]. 

As described above, ZEB1 was identified as a downstream target of FOXC2 [201]. ZEB1 is a transcription factor that belongs to the ZEB family of transcription factors. It is distinguished by the presence of two zinc finger clusters, which are important for DNA binding, as well as a homeodomain that is centrally positioned. Other protein binding domains found in ZEB1 include the Smad interaction domain (SID), the CtBP interaction domain (CID), and the p300-P/CAF binding domain, among others (CBD). ZEB1 can bind to certain DNA sequences known as E-boxes and either downregulate or upregulate the expression of its target gene by recruiting co-suppressors or co-activators through the CID, SID, or CBD signaling pathways. The suppression of ZEB1 in MDA-MB-231 human breast cancer cells results in the overexpression of around 200 genes and the downregulation of approximately 30 genes, the majority of which are determinants of epithelial differentiation and cell–cell adhesion. Because of the critical function of ZEB1 in the downregulation of E-cadherin, it is thought to operate as a driver of the EMT and cancer progression. In addition to suppressing the expression of E-cadherin, ZEB1 regulates the expression of several additional target genes that are implicated in tumor growth. For instance, ZEB1 binds to the promoters of epithelial polarity genes and suppresses their transcription, causing breast cancer cells to lose adherence and thus conferring invasive potential [202].

P38 can activate HIF-1 by stabilizing its α subunit (HIF-1α). HIF-1 is also a transcriptional regulator of growth factors and cytokines such as VEGF and TGF-β that are involved in EMT. In addition, HIF-1 can directly stimulate the production of SNAI and TWIST affecting cell migration and EMT [203,204]. P38α also may trigger cell migration or cytoskeletal remodeling via the phosphorylation of heat-shock protein 27 (HSP27), the activation of LIM domain kinase 1 (LIMK1), and the inactivation of cofilin [205,206]. Cofilin is a small abundant protein that binds both G-actin (monomeric) and F-actin (filamentous actin) and thus confers cell migration. Several studies have found that the expression of specific genes in the cofilin pathway is altered in invasive tumor cells, suggesting that cofilin is involved in the initiation of the early phases of the motility cycle. Moreover, the cofilin pathway responds to the TME stimuli that are implicated in cell migration through the activation of other pathways (see Section 5) involved in metastasis. These include cytokines and growth factors such as EGF and TGFα [207].

P38 responds to ROS buildup by encouraging growth stagnation and death, hence preventing carcinogenesis. TNF-α can be stimulated by ROS, resulting in the activation of the JNK signaling pathway and the induction of apoptosis. On the other hand, TNF-α may activate NF-κB and decrease ROS production through the induction of associated genes such as manganese superoxide dismutase (MnSOD) and ferritin heavy chain (FHC), blocking JNK activation and apoptosis. The activation of P38 stimulates the activity of ribosomal protein S6 kinase α 5/4 (RPS6KA5/4 or MSK1/2) and, in turn, promotes the activity of NF-κB [70]. More recently, plasma membrane Ca^2+^ pump isoform 4b (PMCA4b or ATP2B4) has been established as a metastasis suppressor in BRAF mutant melanoma cells. The activation of P38 triggers the endo/lysosomal internalization and degradation of the ion pump in melanoma cells. Moreover, the inhibition of the P38 MAPK pathway reduces both migration, and metastasis of BRAF mutant cells via the increase in PMCA4b expression and a reduction in β4 integrin yields [208]. 

**Figure 5:**
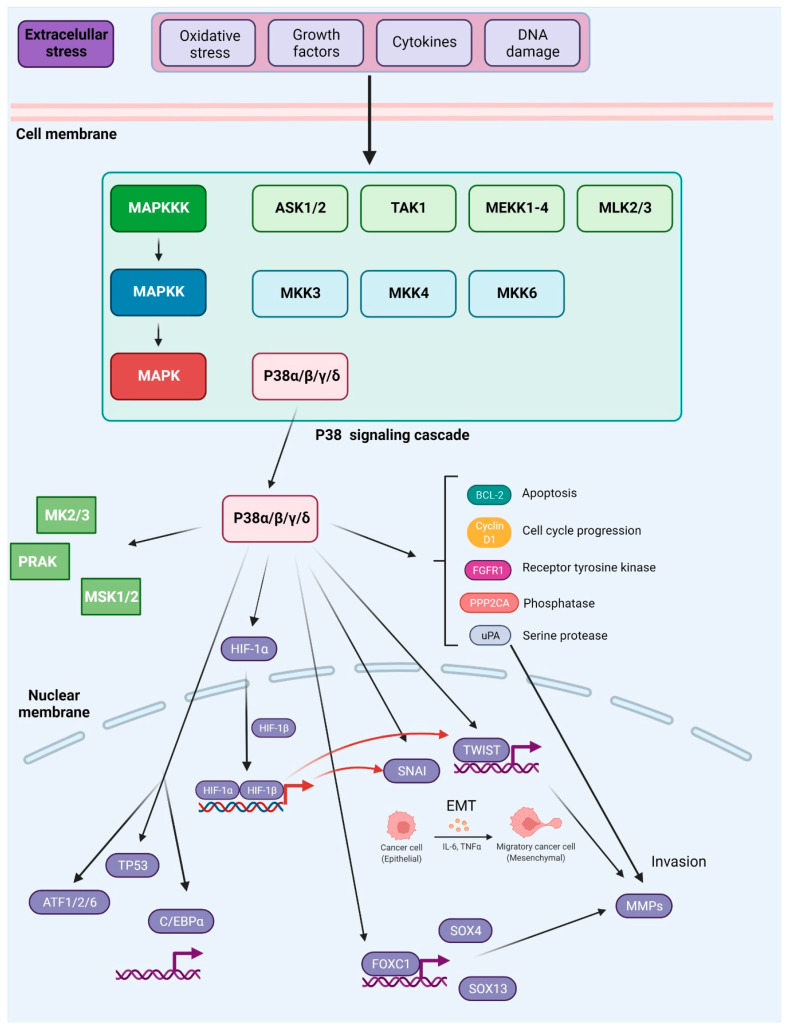
Activation and downstream targets of P38 MAPK. P38 MAPK is activated through several mechanisms. The canonical MAPK signaling module involves sequential phosphorylation and activation events that pass down from MAP3Ks to MAP2Ks, and from MAP2Ks to P38 MAPK. In response to various external stresses and signals (e.g., oxidative stress, UV irradiation, DNA-damage chemotherapeutic agents, and cytokines), several MAP3Ks can trigger activation of P38 signaling, such as TAK1, MEKK1-4, MLK2/3, and ASK1/2. Three MAP2Ks, namely MKK3, MKK6, and MKK4, are direct upstream activators of P38 MAPK. In addition to canonical activation, P38a, the best-characterized member of the P38 kinase family, can also be activated through autophosphorylation. P38 MAPK has been reported to phosphorylate more than 100 proteins, highlighting the versatility of this signaling pathway. Prominent downstream targets include transcription factors, protein kinases, and phosphatases, growth factor receptors, as well as key regulators of cell cycle and apoptosis (depicted in the main text of the article). Based on [181].

While other isoforms of P38 were shown to have a profound influence on cancer metastasis, for many years P38δ was a poorly investigated member of the MAPK family. However, it was found that this isoform is highly expressed in particularly all types of human breast cancers, and the inhibition of P38δ in MCF-7 and MDA-MB-231 breast cancer cell lines results in diminished cell proliferation. Moreover, cells without P38δ seem to exhibit enhanced cell–matrix adhesion. This is attributed to the regulatory role of P38δ on FAK kinase [209]. Moreover, P38δ was shown to enhance the development of CSCs in breast cancer [210]. In contrast, P38γ and P38δ activation may suppress CSCs development in non-small-cell lung cancer (NSCLC) through promotion of the ubiquitin-mediated degradation of SOX2, OCT4, NANOG, KLF4 and MYC transcription factors that normally contribute to the acquisition of cancer stem cell characteristics [211]. The role of P38 signaling in metastasis was also previously summarized in [180] and is shown in Figure 5.

JNK1, JNK2, and JNK3 are the kinases encoded by genes belonging to the JNK family. JNK1 and JNK2 are expressed throughout the body, whereas JNK3 expression is restricted to certain tissues, with the highest levels found in the brain, heart, and testes. For each of the genes, several different splice variants result in a total of 10 isoforms of the protein with molecular weights ranging from 46 to 54 kDa [212]. MKK4 and MKK7 are two representatives of the MAPKK kinases belonging to the JNK sub-pathway activated when MAPKKKs are triggered. These components then phosphorylate and activate JNK, which in turn phosphorylates a multitude of substrates of the AP-1 transcription factor, with c-JUN, FOS, and FOS-related antigen 1/2 (FRA1/2) being the most relevant. Other JNK’s downstream targets include members of the mitochondrial apoptosis regulator BCL-2 family (BCL-2, BCL-xL, BAD, BIM, and BAX), as well as ATF2, ELK-1, TP53, and c-MYC [213,214]. 

JNKs have a dualistic role in cancer [215]. For example, in mouse embryonic fibroblasts (MEFs), the deletion of JNK2 results in increased cell proliferation, whereas the loss of JNK1 has the opposing effect [216]. The differential regulation of c-JUN is thought to be responsible for these effects [217]. In MEFs, JNK loss in combination with the double knockout of TP53 (TP53–/–) results in MET, as demonstrated by increased E-cadherin expression, decreased N-cadherin expression, and lower colony-forming ability [218]. TGFβ activates JNKs in a cascade that necessitates the involvement of TAK1. This pathway is critical for TGFβ signaling because it is required for the phosphorylation of SMAD3 by JNK and is required for the subsequent transcriptional activation of SMAD3. Not only does the phosphorylation of SMAD3 by JNK increase the efficacy of SMAD-dependent gene expression, but it also increases SMAD3 translocation to the nucleus. Because SMAD3 directly transactivates SNAI1 and SNAI2, JNKs may promote the EMT [212]. 

Furthermore, the role of JNKs in inflammation is well established [219,220,221]. Activated JNK1 promotes the recruitment of inflammatory macrophages, which release VEGF to stimulate angiogenesis and MMPs to aid in tissue remodeling. Moreover, monocytes release TGF-β, which in turn causes tumor cells to undergo the EMT [212]. Studies suggest that the double knockout JNK1−/− results in reduction in tumor burden, tumor proliferation, and cytokine production, including TNFα and IL-6. Several studies have suggested that the JNK-dependent inflammatory response promotes tumor progression through induction of the EMT in cells [222]. Moreover, JNK-stimulated binding of c-JUN to the VEGF promoter may increase the expression of angiogenic factors facilitating the access of tumor cells to oxygen and nutrients [223,224]. Phosphorylated JNK activates c-JUN, which results in an increase in the expression of MMP2 as a result of the upregulation of astrocyte elevated gene-1 (AEG-1) in cells. The upregulation of AEG-1 dramatically increases the aggressiveness of osteosarcoma cells via the JNK/c-JUN/MMP2 pathway. In addition, it has been shown that the JNK pathway can promote cancer invasion and metastasis by boosting the expression of other MMP family members such as MMP7 and MMP9, which are induced by the activation of the downstream signaling cascade [213]. 

In human cancer, it is common to observe apparent defects in cell polarity. The fundamental processes through which cell polarity disturbance contributes to tumor growth and metastasis are uncertain. When different apicobasal polarity genes in *Drosophila* are mutated, JNK signaling is activated and the E-cadherin/β-catenin adhesion complex is downregulated. Both of these events are required and sufficient to cause oncogenic RAS(V12)-induced benign tumors in the developing eye to exhibit metastatic behavior. Furthermore, when oncogenic RAS is present, active JNK and RAS signaling work together to promote tumor development, with JNK signaling switching from a proapoptotic to a pro-growth function depending on the context [225]. 

The overexpression of glucose-regulated protein 94 (GRP94) has been observed in a variety of malignancies, including breast, liver, lung, colorectal, gastric, pancreatic, and head and neck cancers. GRP94 is a key protein involved in mediating cancer progression, and it is highly expressed in hepatocellular carcinoma (HCC). On the other hand, chaperonin-containing TCP1 complex (CCT1-8) proteins are highly conserved molecular chaperones that are involved in promoting the correct folding of newly synthesized proteins or the refolding of misfolded proteins. Furthermore, it has been proposed that CCT proteins are implicated in the progression of a variety of cancers, including breast cancer, colorectal cancer, uterine sarcoma, and lung cancer, among others. CCT8 overexpression has been discovered in a variety of cancers, including colon cancer, breast cancer, glioma, and HCC. It has been reported that the silencing of GRP94 hindered the wound healing, migration, and invasion of HCC cells. These findings suggested that GRP94 knockdown may have a suppressive impact on HCC cell metastasis via a reduction in CCT8/c-JUN/EMT signaling in HCC cells. The silencing of GRP94 greatly reduced the migration and invasion of cells [226]. 

JNK is a multifunctional protein that can mediate both cell transformation and apoptosis through a variety of mechanisms that partially overlap with those of the ERK signaling pathway. JNK has been shown to increase resistance to ERK pathway inhibitors as well as chemotherapeutic agents. Moreover, JNK is unquestionably significant in the development of resistance to RAF inhibitors [159]. Evidence suggests that nuclear apoptosis-inducing factor 1 (NAIF1), a protein often downregulated or lost in cancer regulates cellular migration and invasion through the MAPK pathway. The human NAIF1 gene encodes a 327-amino acid protein with a homeodomain-like region and two nuclear localization signals at its N-terminus. The overexpression of NAIF leads to cell growth inhibition and apoptosis. GC cell growth, migration, and invasiveness can be suppressed by NAIF1. NAIF1 can decrease the expression MMP2 and MMP9, and reduce the activity of FAK. Additionally, NAIF1 restrains MAPK1 and MAPK8 activity via the inhibition of their mRNA expression with accompanied ERK and JNK degradation. Thus, the therapeutic targeting of NAIF1 seems to be a new potential strategy in GC treatment [227,228]. Moreover, JNK1 contributes to the survival of circulating cancer cells via inhibition of the transcription of apoptosis-inhibiting genes. As a result, JNK1 and JNK2 may work in concert to improve CTC survival by boosting survival signals and inhibiting apoptosis [212].

Transgelin is an actin-binding protein that is involved in the promotion of cell motility in healthy cells. Although there is debate over whether or not transgelin plays a role in cancer development, many studies have demonstrated that elevated transgelin levels are associated with aggressive tumor behavior, advanced stage of the disease, and poor prognosis [229,230]. Changes in the expression of the transgelin protein mediated by the AKT and JNK signaling pathways increase the metastatic potential of CRC cells. The suppression of transgelin, AKT, or JNK signaling results in a significant reduction in cell migration and invasion in SW620 cells with the concurrent inhibition of actin cytoskeleton dynamics [231]. 

It has been observed that tenascin-C (TNC), an extracellular matrix glycoprotein, may influence metastases and contribute to the poor prognosis of patients with pancreatic cancer. TNC was shown to induce the migration and invasion of pancreatic cancer cells. This was associated with the upregulation of EMT-associated markers, including MMP9, in a JNK/c-JUN-dependent manner. Moreover, because TNC can activate JNK, it can enhance the association of paxillin with FAK, which promotes pancreatic cancer cell motility and adhesion [232]. The role of JNKs in metastasis was summarized elsewhere [212]. 

Several investigations have demonstrated that JNK is involved in the migration and invasion of prostate cancer cells. In PC3 and DU145 cells, the inhibition of JNK pathways by the JNK inhibitor SP600125 or JNK siRNA prevented thrombospondin-2-induced migration and invasion [233]. It has been also demonstrated that the CC chemokine receptor 7 (CCR7) may significantly boost the expression of phosphorylated JNK in PC3 cells by activating NOTCH signaling. This results in increased migration and enhanced metastatic activity in PC3 cells [234].

ERK signaling events are tightly controlled cascades. These regulatory components include bispecific phosphatases, scaffold proteins, control of signal duration, and intensity, as well as the dynamic subcellular localization of cascade components in response to environmental stimuli [126]. More recently, MLK3 has been identified as a crucial player in MAPK signaling with an impact on cell invasion and metastasis. MLK3 belongs to the class of MAPKKK that transduce signals from cell surface receptors to JNK, ERK, and P38 kinases. In mammals, MLK comprises four members: MLK1 (MAP3K9), MLK2 (MAP3K10), MLK3 (MAP3K11), and MLK4 with two isoforms (MLK4α and MLK4β). It is essential for migrating cells to undergo cytoskeletal rearrangement and FA changes, which are controlled both spatially and temporally by the activities of the GTPases CDC42, RAC1, and RHOA. MLK3 works as a scaffold protein for RAF1 and allows subsequent BRAF phosphorylation and activation of MEK1/2 and ERK. MLK3 acts as a negative regulator of RHOA GTPase via direct binding to RHOA-specific guanine exchange factor P63RHO-GEF [235]. In breast cancer cells, the catalytic activity of MLK3 is essential for the activation of JNK, which in turn phosphorylates Ser178 of paxillin, resulting in the proliferation of the cancer cells. This phosphorylation event on paxillin engages FAKs, which in turn stimulates further phosphorylation of paxillin on Tyr31 and Tyr118 [236]. Phosphorylated paxillin is capable of competing with the RHOA-specific GAP protein, P190RHO-GAP, for binding to the P120RAS-GAP. In this way, when paxillin attaches to P120RAS-GAP, it releases P190RHO-GAP from the binding site of P120RAS-GAP, allowing P190RHO-GAP to decrease the activity of RHOA [237]. In addition to being essential for optimal JNK activation. MLK3 distribution in the centrosome and on microtubules during mitosis appears to govern microtubule structure in a JNK-independent manner [238]. Several members of the JNK-interacting proteins (JIPs), including JIP-1, -2, and 3, have been shown to function as scaffold proteins for the MLK3-MKK7-JNK signaling subsystem. It has been demonstrated that the JIP-2 protein serves as a docking site for the recruitment of MLK3, MKK3, and either the P38α or P38δ isoforms of MAPK, allowing for MLK3-dependent P38 MAPK activation to occur more efficiently. MLK3 has been demonstrated to signal through a variety of receptors, including EGFR [45], and the discoidin domain receptor 1 (DDR1) [83]. As a result, downstream JNK, ERK, or P38 signaling is triggered. MLK3 has been also shown to be involved in the invasion of triple-negative breast cancer (TNBC) cells triggered by C-X-C chemokine receptor type 4 (CXCR4)/stromal cell-derived factor 1 (CXCL12). Highly metastatic TNBC cells can be prevented from migrating by inhibiting either the MLK3 or JNK pathways, or by silencing the MLK3 gene. In highly invasive breast cancer cells, the depletion of MLK3 or suppression of its activity leads to increased RHOA activity, excessive FA and stress fiber production, and as a result, reduced cell motility. One possible mechanism by which MLK3 may govern cancer cell invasion is through the regulation of the expression of MMPs. For example, the expression of MMP2 and MMP9 is dependent on the MLK3–ERK–AP1 axis. This suggests that MLK3 may promote cancer invasion in part by upregulating MMPs. Furthermore, MLK3 promotes the EMT switch triggered by collagen type I in prostate cancer. In this model, MLK3 transduces signaling from two collagen receptors, the integrin 2 and the DDR1 receptors, increasing the production of the EMT marker N-cadherin in a process mediated through the MKK7-JNK pathway [239]. The function of MLK3 in proliferation, invasion, and metastasis was reviewed in [239]. 

## 9. Conclusions

Metastasis is perhaps the most common reason for treatment failure in cancer patients, as well as the leading cause of cancer-related death. Even though the emergence of technological breakthroughs in imaging and cancer cell identification that have significantly advanced our understanding of cancer metastasis, the biological mechanisms driving cancer metastasis and chemoresistance remain largely unclear [240]. The understanding of the complicated biology underpinning the survival strategy of cancer cells, especially CSCs following treatment with anticancer agents, is of particular importance. The understanding of signaling pathways involved in the acquisition of metastatic phenotype such as Hedgehog [71,91], NOTCH [92,93], WNT signaling [94,95], and pathways engaged in the proliferation and survival of cancer cells such as those exerted via stimulation of RTKs offers an opportunity to target cancer cells. Furthermore, cancer cells reprogram their metabolism to survive in the harsh conditions that they experience during metastatic processes. The understanding of the mechanisms that underlie metabolic reprogramming in cancer cells may aid the identification of anticancer targets [241,242]. Despite the emergence of novel small-molecule inhibitors that target critical components of the aforementioned signaling pathways, only a small fraction has been tested in clinical settings due to their non-specific toxicity and solubility difficulties that limit their practical application. This could be resolved by nanoformulating these substances, which will help to overcome these barriers and transport molecules to the designated areas in a more targeted manner [243]. 

As evidenced by an increase in the number of publications released each year, it is becoming increasingly clear that MAPKs are involved in all the steps required for hyperproliferating cells to develop into metastatic tumors. However, we are currently lacking in vivo data to fully understand how MAPK signaling pathways can affect the progression of metastatic disease. The understanding of interplay between distinct members of the MAPK family, as well as their crosstalk with other signaling pathways, miRNAs, and long non-coding RNAs is crucial. It is hoped that this research will result in a detailed characterization of the function of MAPK pathway members in the tumor microenvironment and their influence on its components that affect the metastatic process, such as tumor-associated macrophages (TAMs) and cancer-associated fibroblasts (CAFs). The generation of genetically engineered animals will allow the manipulation and, therefore, understanding of individual functions of pathway components in the invasion and metastasis of cancer cells. Most significantly, the knowledge that is collected may be applied to the development of novel therapeutic strategies with fewer side effects and higher therapeutic efficiency. 

## Data Availability

Not Applicable.

## Abbreviations

4E-BP1	Eukaryotic translation initiation factor 4E-binding protein 1
ADCs	Antibody–drug conjugates
AEG-1	Astrocyte elevated gene-1
AKT	RAC-alpha serine/threonine-protein kinase
ANCs	Antibody–nanoparticle conjugates
ARP2/3	Arp2/3 complex 34 kDa subunit
ASK1	Apoptosis signal-regulating kinase 1
ATF2	Cyclic AMP-dependent transcription factor 2
BCL-2	Apoptosis regulator Bcl-2
BCL-xL	Antiapoptotic protein BCL-xL
BM	Basement membrane
BRAF	Serine/threonine-protein kinase B-raf
CAF	Cancer-associated fibroblasts
CBD	p300-P/CAF binding domain
CCT	Chaperonin-containing TCP1 complex
C/EBPα	CCAAT/enhancer-binding protein alpha
CASP8	Caspase 8
CCDC134	Coiled-coil domain-containing protein 134
CD31	Vascular endothelial cluster of differentiation 31
CDC25A	M-phase inducer phosphatase 1
CDC42	Cell division control protein 42 homolog
CDK	Cyclin-dependent kinases
CDKN2A	Cyclin-dependent kinase inhibitor 2A
CID	CtBP interaction domain
COX2	Cyclooxygenase-2
CRC	Colorectal cancer
CSCs	Cancer-stem cells
CTCs	Circulating tumor cells
CXCL12	Stromal cell-derived factor 1
CXCR4	C-X-C chemokine receptor type 4
DDR1	Discoidin domain receptor 1
DIA1	Formin diaphanous 1
DNMT1	DNA methyltransferase 1
ECM	Extracellular matrix
EGF	Epidermal growth factor
EGFR	Epidermal growth factor receptor
eIF-4E	Eukaryotic translation initiation factor 4E
ELK-1	ETS domain-containing protein
EMT	Epithelial–mesenchymal transition
ERK1/2	Extracellular signal-regulated kinase 1/2
ESR	Estrogen receptor
FA	Focal adhesions
FAK	Focal adhesion kinase
FGFR1	Fibroblast growth factor receptor 1
FHC	Ferritin heavy chain
FOX	Forkhead box
FOXC1/2	Forkhead box protein C1/2
FRA1/2	FOS-related antigen 1/2
FYN	SRC family tyrosine kinase FYN
GAPs	GTPase-activating proteins
GBC	Gallbladder cancer
GC	Gastric cancer
GDIs	Guanine nucleotide dissociation inhibitors
GEFs	Guanine nucleotide exchange factors
GLI2	Glioma-associated oncogene homolog 2
GLS	Glutaminase
GLUL	Glutamine synthetase
GLUT	Glucose transporters
GPCRs	G protein-coupled receptors
GRB2	Growth factor receptor-bound protein 2
GRP94	Glucose-regulated protein 94
HCC	Hepatocellular carcinoma
HDAC	Histone deacetylase 1
HER2	Human epidermal growth factor receptor 2
HIF1α	Hypoxic-responsive transcription factor α
HNRNPA1	Heterogeneous nuclear ribonucleoprotein A1
HRE	HIF-responsive element
HSP27	Heat-shock protein 27
IL	Interleukin
I-SMAD	Inhibitory SMADs
JIPs	JNK-interacting proteins
JNK	c-Jun N-terminal kinase
KRAS	GTPase KRas
LAT1	Large neutral amino acid transporter 1
LIMK1	LIM domain kinase 1
MAP	Microtubule-associated protein
MAPK	Mitogen-activated protein kinase
MAPKK	Mitogen-activated protein kinase kinase
MAPKKK	Mitogen-activated protein kinase kinase kinase
MCT	Monocarboxylate transporters
MEF	Mouse embryonic fibroblasts
MEK1	MAPK/ERK kinase 1
MEKK1	Mitogen-activated protein kinase kinase kinase 1
MET	Mesenchymal–epithelial transition
MK2/3	MAP kinase-activated protein kinase 2/3
MLC2	Myosin II light chain
MLCP	Myosin-light-chain phosphatase
MLK3	Mitogen-activated protein kinase kinase kinase 11
MMPs	Matrix metalloproteinases
MNK	MAP kinase interacting kinase
MnSOD	Manganese superoxide dismutase
MRCK	Myotonic dystrophy kinase-related Cdc42-binding kinase
MSK1	Ribosomal protein S6 kinase alpha-5
mTOR	Serine/threonine-protein kinase mTOR
MYC	MYC proto-oncogene protein
NAIF1	Nuclear apoptosis-inducing factor 1
NCAM	Neural cell adhesion molecule
NFκB	Nuclear factor kappa B subunit
NHE	Sodium hydrogen ion exchangers
PDGF-D	Platelet-derived growth factor D
PDGFR	Platelet-derived growth factor receptor
PDK1	3-Phosphoinositide-dependent protein kinase 1
PECAM	Platelet and endothelial cell adhesion molecule 1
PI3K	Phosphatidylinositol-4,5-bisphosphate 3-kinase
PIK3CA	Phosphatidylinositol-4,5-bisphosphate 3-kinase catalytic subunit alpha
PKC	Protein kinase C
PLC	Phospholipase C
PPP2CA	Serine/threonine-protein phosphatase 2A catalytic subunit alpha isoform
PRL3	Protein tyrosine phosphatase type IVA 3
PRRX1	Paired mesoderm homeobox protein 1
PTEN	Phosphatase and tensin homolog
PMCA4b	Plasma membrane Ca^2+^ pump isoform 4b
PTHrP	Parathyroid hormone-related protein
R-SMAD	Receptor-activated SMADs
RAP1A	Ras-related protein Rap-1A
RAC1	Ras-related protein RAC1
RAF	Proto-oncogene serine/threonine-protein kinase RAF
RANK	Tumor necrosis factor superfamily member 11
RANKL	Tumor necrosis factor ligand superfamily member 11
RASAL1	RasGAP-activating-like protein 1
RB	Retinoblastoma protein
RHOA	Transforming protein RhoA
RHOE	RHO-related GTP-binding protein E
ROCK	RHO-associated kinase
ROS	Reactive oxygen species
RPS6KA5/4	Ribosomal protein S6 kinase alpha-5/4
RTK	Receptor tyrosine kinase
S6K	Ribosomal protein S6 kinase
SID	Smad interaction domain
SHCA	SRC homology 2 domain-containing-transforming A
SFPQ	Polypyridine tract-binding protein-associated splicing factor
SLC4/6	Sodium-coupled bicarbonate transporters
SMAD	Mothers against decapentaplegic
SNAT2	Sodium-coupled neutral amino acid transporter 2
SOS	Son of sevenless
SPRY2	Sprouty 2
STAT3	Signal transducer and activator of transcription 3
TAK1	Transforming growth factor-β-activated kinase 1
TAM	Tumor-associated macrophage
TCA	Tricarboxylic acid
TGFα/β	Transforming growth factor alpha/beta
TICs	Tumor-initiating cells
TIMPs	Tissue inhibitors of metalloproteinases
TME	Tumor microenvironment
TNBC	Triple-negative breast cancer
TNC	Tenascin-C
TNFα	Tumor necrosis factor alpha
TP53	Cellular tumor antigen p53
TRAF6	TNF receptor-associated factor 6
TWIST1	Twist-related protein 1
TβR1/2	TGF-β type receptor 1/2
uPA	Urokinase plasminogen activator
V-ATPase	Vacuolar-type H+-adenosine triphosphatases
VEGF	Vascular endothelial growth factor
VEGFR	Vascular endothelial growth factor receptor
VHL	Von Hippel–Lindau tumor suppressor
WASP	Wiskott–Aldrich syndrome protein
ZEB1/2	Zinc finger E-box-binding homeobox 1/2
ZIC1	Zinc finger protein ZIC1
